# Knowledge and attitudes towards Alzheimer’s disease among adult residents in Jiulongpo, Chongqing, China: A cross-sectional survey

**DOI:** 10.1371/journal.pone.0327346

**Published:** 2025-06-30

**Authors:** Jinhua Zhang, Yao Huang, Yi Jiang, Tong Li

**Affiliations:** 1 Department of Non-communicable Chronic Disease Control and Prevention, Disease Control and Prevention Center of Jiulongpo District, Chongqing, China; 2 Research Center for Medicine and Social Development, School of Public Health, Chongqing Medical University, Chongqing, China; Kennesaw State University, UNITED STATES OF AMERICA

## Abstract

**Background:**

The prevalence of Alzheimer’s disease (AD) continues to rise as the aging population increases, and the resulting cognitive decline severely affects patients’ quality of life. However, the public’s understanding of AD is markedly insufficient, which limits the effectiveness of early prevention and treatment. This study conducted a cross-sectional survey to assess the level of knowledge about AD among adult residents in Jiulongpo District, Chongqing, as well as their attitudes towards people with dementia, providing a basis for formulating targeted intervention strategies.

**Methods:**

A cross-sectional survey was conducted in Jiulongpo, Chongqing, China, from September to October 2024. The self-reported questionnaire for participants included three parts: demographic information, the Alzheimer’s Disease Knowledge Scale (ADKS), and the Dementia Attitude Scale (DAS). Multiple linear regression analysis was used to identify the main factors influencing AD knowledge and attitudes.

**Results:**

1,362 respondents were surveyed, with 64.24% female. The age group of 40–64 has the largest number of people, accounting for 38.03%. The average ADKS score was 17.67 (standard deviation = 2.47), with individual item score rates ranging from 16.01% to 89.72% across the 30 items. The domains with the lowest ADKS score rates were “Caregiving” (43.40%) and “Symptoms” (52.25%). The average score on the DAS was 81.72 (standard deviation = 11.19). The score rates for the “Dementia knowledge,” “Positive social comfort,” and “Negative social comfort” dimensions of the DAS were 61.56%, 55.02%, and 56.38%, respectively. Residents aged 18–39, living in urban areas, with higher education or above, working in healthcare, having a higher income level, being unmarried, and having experience in Alzheimer’s Disease and Related Dementias (ADRD) knowledge training have a higher level of knowledge about AD and a more positive attitude towards it. Residents with family members suffering from ADRD have lower DAS scores than those without such family members.

**Conclusion:**

People’s understanding of AD is limited, especially in the areas of “Caregiving” and “Symptoms”, and they hold a neutral attitude toward dementia. We recommend intensifying health education efforts on AD knowledge across different populations, particularly emphasizing the promotion of knowledge in weak areas, while also cultivating their friendly attitudes towards dementia patients.

## Introduction

As global aging intensifies, the prevalence of Alzheimer’s disease (AD) and related cognitive disorders is also gradually increasing. In 2017, there were approximately 50 million dementia patients worldwide [1]. By 2020, the number of dementia patients globally exceeded 55 million, with the majority of this growth coming from developing countries [2]. It is estimated that this figure will grow to over 131 million by 2050 [3].

According to a national cross-sectional study conducted in 2020, the prevalence of dementia among people aged 60 and above in China is 6.0%, with AD accounting for approximately 65% of these cases [4]. The “World Dementia Report” (2021) released by the World Health Organization (WHO) indicates that China has the largest number of dementia patients in the world [5]. The Chinese government and society are paying increasing attention to Alzheimer’s disease and related cognitive impairments. In 2021, the Alzheimer’s Disease Prevention and Control Association of China released the “Strategic Action Plan for China to Address AD,” proposing systematically carrying out universal science popularization education to enhance public awareness and participation. In 2023, the National Health Commission of China issued the “Notice on Implementing the Dementia Prevention and Promotion Action (2023-2025),” explicitly stating the need to intensify the promotion of scientific knowledge on dementia prevention and control, and continuously improve public awareness of dementia prevention and control knowledge, to create a favorable atmosphere in the whole society for caring for elderly people with dementia. In January 2025, the “National Action Plan for Addressing Dementia in Later Life (2024-2030)”, jointly issued by 15 departments including the National Health Commission of China, stated that by 2030, scientific knowledge on dementia prevention and control will be popularized, with the public awareness rate of dementia prevention and control knowledge reaching 80% or above. Targeted science popularization and health education are essential to achieve this expected goal, and the premise for this is to understand the current level of public awareness and attitudes toward dementia.

Numerous studies have examined knowledge and attitudes towards AD among specific groups. For instance, Wang conducted a cross-sectional survey among community health professionals in Changsha, China [6]. Similarly, Qiu investigated the knowledge of dementia among community doctors from all provinces across China [7]. Wang also carried out similar research among nursing and medical students at four universities in China [8]. Additionally, Yang assessed the current levels of knowledge, attitudes, and health behaviors regarding AD among residents in communities in Tianjin, China, and identified the factors associated with these attributes [9]. Furthermore, Zuo’s research aims to investigate and classify the level of knowledge about AD among nursing staff in the eastern China region, and to identify the factors that influence their knowledge level of AD [10]. Overall, the majority of studies have been conducted on groups with a medical background, while there have been fewer studies targeting the general adult population [6–10]. Moreover, due to differences in economic development levels, education levels, regional cultures, and sample representativeness across various regions in China, the results of the aforementioned studies have limited targeted guidance for the Jiulongpo District in Chongqing.

Understanding the public’s knowledge level of AD helps to assess the effectiveness of current health education efforts and guide the design of future intervention measures, thereby increasing people’s willingness to undergo early screening and diagnosis [11, 12]. At the same time, understanding the public’s attitudes toward AD is crucial for implementing socio-psychological treatment measures [13]. Therefore, it is essential to conduct regional research on the knowledge and attitudes of residents towards AD, to develop more targeted prevention and control measures, and thereby enhance the public’s awareness and friendliness towards dementia.

## Methods

### Research design and participants

The study employed a descriptive cross-sectional survey design. Conducted from September to October 2024 in Jiulongpo District, Chongqing, this research aims to assess the knowledge and social attitudes towards AD among residents aged 18 and above in Jiulongpo District.

### Sample size calculation

The sample size for this study was determined based on the formula for cross-sectional surveys: $\;n=\frac{Z²p(1-p)}{d²}$ [14]. Where Z represents the 95% confidence interval, with a value of 1.96. The P-value is the assumed awareness level, which, based on the literature, is set at 60%. d denotes the acceptable margin of error, set at 2%. Considering factors such as refusal rates and questionnaire validity, we increased the sample size by 10% on this basis, resulting in an anticipated total of approximately 1,268 participants. The final count of valid participants who completed the survey reached 1,362.

### Measurements

The survey utilized a three-part questionnaire: socio-demographic information, the Alzheimer’s Disease Knowledge Scale (ADKS), and the Dementia Attitudes Scale (DAS). All contents were self-administered by the participants.

The sociodemographic information includes gender, age, types of residence, education level, marital status, occupation, monthly income, family members with Alzheimer’s Disease and Related Dementias (ADRD), caregiving experience for ADRD, and knowledge training experience related to ADRD.

The ADKS, developed by Carpenter et al. in 2009 [15], is suitable for assessing the knowledge of AD among different populations. The Chinese version of the ADKS was adapted by He Runlian et al. [16], with a Cronbach’s α coefficient of 0.756. The scale covers seven domains: risk factors, assessment and diagnosis, symptoms, disease course, life impact, caregiving, and treatment management, with 30 items. A correct response scores 1 point, while an incorrect response scores 0 points. The total score ranges from 0 to 30, with higher scores indicating greater knowledge of AD.

The DAS was developed by O’Connor et al. in 2010 as a tool to assess attitudes towards dementia [17]. The Chinese version of the DAS was introduced and adapted by Li Huanli et al [18]. The scale covers three dimensions: dementia knowledge, positive social comfort, and negative social comfort, with a total of 20 items. It uses a Likert 7-point scoring system, where “completely disagree” is scored as 1 and “completely agree” is scored as 7. The 6 items in the negative social comfort dimension are scored in reverse. The total score on the scale ranges from 20 to 140, with higher scores indicating more positive attitudes toward dementia.

### Statistical analysis

Statistical analysis was conducted using IBM SPSS Statistics v.26.0 (IBM, Armonk, NY, United States). Descriptive statistics described demographic data, ADKS, and DAS scores. Continuous variables were described as means (M) and standard deviations (SD), while categorical variables were expressed as frequencies (n) and percentages (%). T-tests and one-way ANOVA were employed to compare whether there were significant differences between groups. To identify the relevant factors influencing AD knowledge and attitudes, multivariate linear regression analysis was performed, with statistically significant variables from the univariate analysis serving as independent variables and ADKS scores and DAS scores as dependent variables. The significance level was set at a two-sided 0.05.

### Ethical considerations

To protect the rights and interests of the participants, all data were strictly confidential. This study was reviewed and approved by the Ethics Committee of the Jiulongpo District Center for Disease Control and Prevention in Chongqing. Participants were informed that their participation in the study was voluntary and that they could withdraw from the survey at any time without reason during the investigation. They participated in the survey after understanding the content and giving their consent. All participants signed the Informed Consent Form.

## Results

### Sociodemographic characteristics of the residents and differences in ADKS and DAS scores

As shown in Table 1, the age distribution of the participants is relatively even. The gender distribution showed that more than half (64.24%) of the respondents were female. 68.21% of people live in cities. In terms of education, 25.26% were non-literate or had completed primary school, 20.04% had completed junior high school, 13.22% had completed high school, and 41.48% had received higher education. Furthermore, the majority of participants (81.79%) were married. The proportion of unemployed individuals is the smallest (7.05%), while medical and health personnel account for the largest share (21.81%). 5.51% of participants had family members with ADRD. 3.82% reported having experience in caring for individuals with ADRD. 14.46% of participants had attended ADRD knowledge training.

**Table 1:** The sociodemographic characteristics of the residents and their relationship with ADKS and DAS scores (N = 1362).

characteristics	n (%)	ADKS, M ± SD	t-value or F-value	*p-*value	DAS, M ± SD	t-value or F-value	*p-*value
**Gender**			−2.536	0.011^*^		−0.054	0.957
Male	487 (35.76)	17.45 ± 2.50			81.70 ± 11.31		
Female	875 (64.24)	17.80 ± 2.44			81.73 ± 11.13		
**Age(Years)**			32.184	<0.001^*^		21.844	<0.001^*^
18-39	439 (32.23)	18.36 ± 2.49			84.57 ± 12.34		
40-64	518 (38.03)	17.60 ± 2.46			80.16 ± 10.04		
≥65	405 (29.74)	17.03 ± 2.27			80.63 ± 10.71		
**Types of residence**			11.483	0.001^*^		14.144	<0.001^*^
City	929(68.21)	17.83 ± 2.57			82.50 ± 11.03		
Township	433(31.79)	17.34 ± 2.19			80.06 ± 11.36		
**Educational level**			39.157	<0.001^*^		6.529	0.003^*^
Non-literate or primary	344 (25.26)	16.83 ± 2.35			80.80 ± 10.40		
Junior high school	273 (20.04)	17.25 ± 2.08			80.86 ± 9.76		
High school	180 (13.22)	17.43 ± 2.04			79.93 ± 11.86		
Higher education and above	565 (41.48)	18.47 ± 2.60			83.27 ± 11.90		
**Marital status**			7.692	<0.001^*^		4.441	0.004^*^
Unmarried	153 (11.23)	18.36 ± 2.56			84.37 ± 13.41		
Married	1114 (81.79)	17.64 ± 2.45			81.48 ± 10.78		
Divorced	35 (2.57)	17.40 ± 1.85			82.74 ± 12.04		
Widowed	60 (4.41)	16.65 ± 2.52			78.88 ± 11.10		
**Occupation**			17.148	<0.001^*^		8.316	<0.001^*^
Farmer	218(16.01)	16.86 ± 2.27			81.00 ± 11.38		
unemployed individuals	96(7.05)	17.32 ± 2.63			81.78 ± 10.47		
Other workers	280(20.56)	17.54 ± 2.37			80.90 ± 9.60		
Worker, employee, service industry personnel	221(16.23)	18.01 ± 2.47			83.41 ± 11.51		
Medical and health personnel	297(21.81)	18.61 ± 2.59			84.26 ± 12.70		
Retiree	250(18.36)	17.25 ± 2.12			78.74 ± 9.88		
**Monthly income (CNY)**			7.111	<0.001^*^		6.214	<0.001^*^
<999	193 (14.17)	17.07 ± 2.34			81.16 ± 10.22		
1000-2999	479 (35.17)	17.60 ± 2.38			80.15 ± 11.65		
3000-5999	553 (40.60)	17.80 ± 2.54			83.00 ± 11.00		
≥6000	137 (10.06)	18.26 ± 2.50			82.83 ± 11.03		
**Family members with ADRD**			−1.471	0.141		2.019	0.044^*^
Yes	75 (5.51)	18.08 ± 2.61			79.19 ± 11.21		
No	1287 (94.49)	17.65 ± 2.46			81.87 ± 11.18		
**Caregiving experience for ADRD**			−0.116	0.908		1.219	0.223
Yes	52 (3.82)	17.71 ± 2.04			79.87 ± 10.29		
No	1310 (96.18)	17.67 ± 2.49			81.79 ± 11.22		
**Knowledge training experience related to ADRD**			−5.665	<0.001^*^		−3.484	0.001^*^
Yes	197 (14.46)	18.58 ± 2.33			84.28 ± 13.47		
No	1165 (85.54)	17.52 ± 2.46			81.29 ± 10.71		

ADKS: Alzheimer’s Disease Knowledge Scale, DAS: Dementia Attitudes Scale, ADRD: Alzheimer’s Disease and Related Dementias.

M: Mean, SD: Standard Deviation, ^*^: statistical difference.

There were no significant differences in ADKS scores between participants with family members having ADRD and those with ADRD caregiving experience (*p* > 0.05), but there were significant differences with all other variables (*p *< 0.05). Significant differences were found in DAS scores among participants of different ages, types of residence, education levels, marital statuses, occupations, monthly incomes, family members with ADRD, and experience of ADRD knowledge training (*p* < 0.05). However, there were no significant differences in DAS scores based on gender and ADRD caregiving experience (*p* > 0.05).

### The scoring situation of residents on the ADKS

Table 2 describes the ADKS scale. The average ADKS score for participants was 17.67, with a score rate of 58.90%. The percentage of correct answers for ADKS items ranged from 16.01% to 89.72%. The scoring rate for the “risk factors” domain is 54.50%, with the lowest score rate (16.96%) for the incorrect statement “Scientific evidence has proven that mental exercise can prevent the onset of AD.” The scoring rate for the “symptoms” domain is 52.25%, with the lowest score rate of only 18.58% for the incorrect statement “Tremors or shaking of the hands or arms are common symptoms in patients with AD.” The accuracy rate for the “disease course” dimension was 70.50%, and only 29.81% of respondents knew that the item “In rare cases, AD patients can recover” was incorrect. The correct response rate for the “assessment and judgment” section was 66.25%, with 17.11% of people correctly answering the statement: “If sudden memory impairment and confusion occur, it is likely due to AD.” The “treatment management” dimension had a scoring rate of 68.75%, with only 29.88% of residents correctly answering that the statement “Using reminder notes may lead to further decline in the abilities of people with AD” is incorrect. The scoring rate for the “life impact” aspect was 53.67%, with the lowest score rate for the item “Most AD patients live in nursing homes,” which only 33.41% of participants correctly identified as incorrect. The accuracy rate for the “care” section was 43.40%, with the lowest score rate being only 16.01% for the item stating, “When a person with AD repeatedly asks the same question or tells the same story, it is helpful to remind them that they are repeating themselves.” This indicates that only 16.01% of residents understand that this statement is incorrect.

**Table 2:** The correct response rate for ADKS items (N = 1362).

Items (correct answer)	Number of correct answers	Percentages (%)
Risk factors		
2. It has been scientifically proven that mental exercise can prevent a person from getting AD. (False)	231	16.96
13. People in their 30s can have AD. (True)	911	66.89
18. Having high cholesterol may increase a person’s risk of developing AD. (True)	1075	78.93
25. Prescription drugs that prevent AD are available. (False)	403	29.59
26. Having high blood pressure may increase a person’s risk of developing AD. (True)	1094	80.32
27. Genes can only partially account for the development of AD. (True)	1160	85.17
Total score ± SD (out of 6)	3.27 ± 0.96	54.50
Symptoms		
19. Tremor or shaking of the hands or arms is a common symptom in people with AD. (False)	253	18.58
22. Trouble handling money or paying bills is a common early symptom of AD. (True)	1110	81.50
23. One symptom that can occur with AD believes that other people are stealing one’s things. (True)	1133	83.19
30. Most people with AD remember recent events better than things that happened in the past. (False)	357	26.21
Total score ± SD (out of 4)	2.09 ± 0.73	52.25
Disease course		
3. After symptoms of AD appear, the average life expectancy is 6–12 years. (True)	1061	77.90
8. In rare cases, people have recovered from AD. (False)	406	29.81
14. A person with AD becomes increasingly likely to fall down as the disease gets worse. (True)	1196	87.81
17. Eventually, a person with AD will need 24-hour supervision. (True)	1173	86.12
Total score ± SD (out of 4)	2.82 ± 0.76	70.50
Assessment and diagnosis		
4. When a person with AD becomes agitated, a medical examination might reveal other health problems that caused the agitation. (True)	1075	78.93
10. If trouble with memory and confused thinking appear suddenly, it is likely due to AD. (False)	233	17.11
20. Symptoms of severe depression can be mistaken for symptoms of AD. (True)	1083	79.52
21. AD is one type of dementia. (True)	1222	89.72
Total score ± SD (out of 4)	2.65 ± 0.72	66.25
Treatment and management		
9. People whose AD is not yet severe can benefit from psychotherapy for depression and anxiety. (True)	1185	87.00
12. Poor nutrition can make the symptoms of AD worse. (True)	1094	80.32
24. When a person has AD, using reminder notes is a crutch that can contribute to decline. (False)	407	29.88
29. AD cannot be cured. (True)	1053	77.31
Total score (out of 4)	2.75 ± 0.79	68.75
Life impact		
1. People with AD are particularly prone to depression. (True)	1081	79.37
11. Most people with AD live in nursing homes. (False)	455	33.41
28. It is safe for people with AD to drive, as long as they have a companion in the car at all times. (False)	660	48.46
Total score ± SD (out of 3)	1.61 ± 0.80	53.67
Caregiving		
5. People with AD do best with simple instructions given one step at a time. (True)	1032	75.77
6. When people with AD begin to have difficulty taking care of themselves, caregivers should take over right away. (False)	358	26.28
7. If a person with AD becomes alert and agitated at night, a good strategy is to try to make sure that the person gets plenty of physical activity during the day. (True)	972	71.37
15. When people with AD repeat the same question or story several times, it is helpful to remind them that they are repeating themselves. (False)	218	16.01
16. Once people have AD, they are no longer capable of making informed decisions about their own care. (False)	379	27.83
Total score ± SD (out of 3)	2.17 ± 0.89	43.40
Total ADKS score ± SD (out of 30)	17.67 ± 2.47	58.90

AD: Alzheimer’s Disease, ADKS: Alzheimer’s Disease Knowledge Scale, SD: Standard Deviation.

### The scoring situation of residents on the DAS

Table 3 presents the frequency and percentage of respondents’ answers to the 20 items of the DAS. The average score for the DAS was 81.72 (out of 140, SD = 11.19), with a score rate of 58.37%. In the domain of “Knowledge about Dementia”, the average score was 38.78 (out of 63, SD = 10.64), yielding a scoring rate of 61.56%. Only one item in this domain had an average score below 4, which was “People with ADRD can also enjoy life”, with an average score of 3.97. The subdomain of “Positive Social Comfort” had an average score of 19.26 (out of 35, SD = 6.11), resulting in a score rate of 55.03%. All items in this subdomain scored below 4, with relatively lower scores for “I feel comfortable when I am with someone who has ADRD” and “I feel comfortable having physical contact with someone who has ADRD”, averaging 3.82 and 3.81, respectively. The subdomain of “Negative Social Comfort” achieved an average score of 23.68 (out of 42, SD = 6.77), with a score rate of 56.38%. In this domain, four items (Item 8, Item 9, Item 16, and Item 17) had average scores below 4, among which Item 16 (I feel frustrated because I don’t know how to help someone with ADRD) had a relatively lower average score of 3.78.

**Table 3:** The scoring situation of the DAS (N = 1362).

Items n (%)	Strongly disagree	Mostly disagree	Slightly disagree	Neutral	Slightly agree	Mostly agree	Strongly agree	M (SD)
**Dementia knowledge**								
7. Every person with ADRD has different needs.	58(4.20)	88(6.46)	147(10.79)	533(39.13)	222(16.30)	168(12.33)	146(10.72)	4.37(1.50)
10. People with ADRD like having familiar things nearby.	59(4.33)	85(6.24)	128(9.40)	540(39.65)	240(17.62)	169(12.41)	141(10.35)	4.39(1.48)
11. It is important to know the past history of people with ADRD.	52(3.82)	78(5.73)	131(9.62)	518(38.03)	206(15.12)	173(12.70)	204(14.98)	4.53(1.55)
12. It is possible to enjoy interacting with people with ADRD.	61(4.48)	85(6.24)	150(11.01)	577(42.36)	230(16.89)	148(10.87)	111(8.15)	4.26 (1.43)
14. People with ADRD can enjoy life.	92(6.75)	116(8.52)	189(13.88)	578(42.44)	199(14.61)	107(7.86)	81(5.95)	3.97(1.44)
15. People with ADRD can feel when others are kind to them.	64(4.70)	89(6.53)	145(10.65)	567(41.63)	222(16.30)	150(11.01)	125(9.18)	4.28(1.46)
18. I admire the coping skills of people with ADRD.	51(3.74)	77(5.65)	146(10.72)	563(41.34)	252(18.50)	138(10.13)	135(9.91)	4.35(1.42)
19. We can do a lot now to improve the lives of people with ADRD.	52(3.82)	77(5.65)	158(11.60)	578(42.44)	254(18.65)	126(9.25)	117(8.59)	4.29(1.39)
20. Difficult behaviors may be a form of communication for people with ADRD.	44(3.23)	71(5.21)	135(9.91)	601(44.13)	259(19.02)	129(9.47)	123(9.03)	4.35(1.36)
Total score ± SD (out of 63)								38.78 ± 10.64
**Positive social comfort**								
1. It is rewarding to work with people who have ADRD.	145(10.65)	156(11.45)	144(10.57)	554(40.68)	146(10.72)	110(8.08)	107(7.86)	3.85(1.62)
3. People with ADRD can be creative.	123(9.03)	132(9.69)	173(12.70)	535(39.28)	211(15.49)	105(7.71)	83(6.09)	3.90(1.53)
4. I feel confident around people with ADRD.	103(7.56)	147(10.79)	188(13.80)	602(44.20)	169(12.41)	84(6.17)	69(5.07)	3.82(1.43)
5. I am comfortable touching people with ADRD.	104(7.64)	141(10.35)	181(13.29)	630(46.26)	160(11.75)	80(5.87)	66(4.85)	3.81(1.41)
13. I feel relaxed around people with ADRD.	92(6.75)	125(9.18)	190(13.95)	614(45.08)	192(14.10)	88(6.46)	61(4.48)	3.88(1.38)
Total score ± SD (out of 35)								19.26 ± 6.11
**Negative social comfort**								
2. I am afraid of people with ADRD.	122(8.96)	159(11.67)	171(12.56)	574(42.14)	167(12.26)	86(6.31)	83(6.09)	4.20(1.51)
6. I feel uncomfortable being around people with ADRD.	97(7.12)	116(8.52)	202(14.83)	604(44.35)	174(12.78)	91(6.68)	78(5.73)	4.10(1.42)
8. I am not very familiar with ADRD.	69(5.07)	95(6.98)	162(11.89)	578(42.44)	220(16.15)	132(9.69)	106(7.78)	3.82(1.44)
9. I would avoid an agitated person with ADRD.	75(5.51)	120(8.81)	163(11.97)	600(44.05)	202(14.83)	112(8.22)	90(6.61)	3.95(1.43)
16. I feel frustrated because I do not know how to help people with ADRD.	61(4.48)	79(5.80)	161(11.82)	594(43.61)	237(17.40)	130(9.54)	100(7.34)	3.78(1.39)
17. I cannot imagine caring for someone with ADRD.	64(4.70)	95(6.98)	165(12.11)	578(42.44)	240(17.62)	120(8.81)	100(7.34)	3.83(1.41)
Total score ± SD (out of 42)								23.68 ± 6.77
**Total DAS score ± SD (out of 140)**								81.72 ± 11.19

AD: Alzheimer’s Disease, DAS: Dementia Attitudes Scale, ADRD: Alzheimer’s Disease and Related Dementias, SD: Standard Deviation, M: Mean.

### Multiple linear regression analysis of residents’ ADKS scores

The results, as shown in Table 4, indicate that all nine variables are factors influencing the ADKS scores of the surveyed residents (p < 0.05). Specifically, women have an ADKS score that is 0.353 higher than that of men (p < 0.05). Residents aged 18–39 have significantly higher ADKS scores compared to those aged 40 and above (p < 0.001). Residents living in townships have significantly lower ADKS scores than those living in cities (p < 0.05). The ADKS scores of residents with higher education or above are significantly higher than those of residents with other levels of education (p < 0.001). The ADKS scores of unmarried residents are higher than those of residents with other marital statuses (p < 0.05). The ADKS scores of healthcare personnel were significantly higher than those of residents in other occupations (p < 0.05). Residents with a monthly income of less than 999 RMB have lower ADKS scores than those with higher incomes (p < 0.05). Individuals with experience in ADRD knowledge training have significantly higher ADKS scores than those without such experience (p < 0.001).

**Table 4:** Multiple linear regression analysis of ADKS.

Predictor variable	B	SE	t-value	95%CI	*p-*value
**Gender**					
Male (ref)					
Female	0.353	0.139	2.536	(0.080~0.627)	0.011^*^
**Age (Years)**					
18-39 (ref)					
40-64	−0.759	0.157	−4.846	(−1.066~−0.452)	<0.001^*^
≥65	−1.326	0.166	−7.971	(−1.652~−0.999)	<0.001^*^
**Types of residence**					
City (ref)					
Township	−0.485	0.143	−3.389	(−0.766~−0.204)	0.001^*^
**Educational level**					
Higher education and above (ref)					
Non-literate or primary	−1.505	0.245	−6.147	(−1.985~−1.025)	<0.001^*^
Junior high school	−1.042	0.226	−4.603	(−1.487~−0.598)	<0.001^*^
High school	−0.877	0.224	−3.915	(−1.316~−0.437)	<0.001^*^
**Marital status**					
Unmarried (ref)					
Married	−0.718	0.211	−3.397	(−1.132~−0.303)	0.001^*^
Divorced	−0.959	0.459	−2.090	(−1.860~−0.059)	0.037^*^
Widowed	−1.709	0.373	−4.580	(−2.442~−2.442)	<0.001^*^
**Occupation**					
Medical and health personnel (ref)					
Farmer	−1.755	0.214	−8.204	(−2.175~−1.335)	<0.001^*^
unemployed individuals	−1.290	0.282	−4.581	(−1.842~−0.737)	<0.001^*^
Other workers	−1.074	0.200	−5.373	(−1.465~−0.682)	<0.001^*^
Worker, employee, service industry personnel	−0.604	0.213	−2.834	(−1.022~−0.186)	0.005^*^
Retiree	−1.361	0.206	−6.610	(−1.765~−0.957)	<0.001^*^
**Monthly income (CNY)**					
<999 (ref)					
1000-2999	0.529	0.209	2.529	(0.119~0.939)	0.012^*^
3000-5999	0.725	0.205	3.536	(0.323~1.127)	<0.001^*^
≥6000	1.190	0.274	4.345	(0.653~1.728)	<0.001^*^
**Knowledge training experience related to ADRD**					
No (ref)					
Yes	1.065	0.188	5.665	(0.696~1.434)	<0.001^*^

B: unstandardized coefficients, SE: Standard Errors, 95%CI: 95% Confidence Intervals, CNY: Chinese Yuan, ref: reference, ADRD: Alzheimer’s Disease and Related Dementias, ^*^: statistical difference.

### Multiple linear regression analysis of residents’ DAS scores

The results are shown in Table 5, indicating that all nine variables are factors influencing the attitudes of respondents toward dementia (p < 0.05). Residents aged 40–64 and ≥65 had significantly lower DAS scores than those aged 18–39 (p < 0.05). Residents living in townships had significantly lower DAS scores than those living in cities (p < 0.001). Residents who had received higher education or above had significantly higher DAS scores than those with other educational backgrounds (p < 0.05). Married and widowed residents had significantly lower DAS scores than unmarried residents (p < 0.05). The DAS scores of farmers, retirees, and other workers were significantly lower than those of healthcare personnel (p < 0.05). Residents with a monthly income of 3000–5999 RMB had higher DAS scores than those with a monthly income of less than 999 RMB (p < 0.05). Residents with family members suffering from ADRD had lower DAS scores than those without such family members (p < 0.05). Individuals with training experience in ADRD knowledge had significantly higher DAS scores than those without such experience (p < 0.001).

**Table 5:** Multiple linear regression analysis of DAS.

Predictor variable	B	SE	t-value	95%CI	*p*-value
**Age (Years)**					
18-39 (ref)					
40-64	−4.411	0.715	−6.168	(−5.814~−3.008)	<0.001^*^
≥65	−3.940	0.760	−5.187	(−5.430~−2.450)	<0.001^*^
**Types of residence**					
City (ref)					
Township	−2.437	0.648	−3.761	(−3.709~−1.166)	<0.001^*^
**Educational level**					
Higher education and above (ref)					
Non-literate or primary	−2.465	0.761	−3.240	(−3.957~−0.973)	0.001^*^
Junior high school	−2.410	0.820	−2.939	(−4.019~−0.802)	0.003^*^
High school	−3.339	0.952	−3.508	(−5.207~−1.472)	<0.001^*^
**Marital status**					
Unmarried (ref)					
Married	−2.888	0.961	−3.005	(−4.774~−1.003)	0.003^*^
Divorced	−1.623	2.089	−0.777	(−5.721~2.475)	0.437
Widowed	−5.483	1.698	−3.228	(−8.814~2.151)	0.001^*^
**Occupation**					
Medical and health personnel (ref)					
Farmer	−3.259	0.985	−3.309	(−1.873~~3.435)	0.001^*^
unemployed individuals	−2.478	1.297	−1.911	(−2.057~1.857)	0.056
Other workers	−3.359	0.920	−3.652	(0.340~4.475)	<0.001^*^
Worker, employee, service industry personnel	−0.852	0.981	−0.868	(1.327~5.191)	0.385
Retiree	−5.523	0.948	−5.827	(−4.271~−0.257)	<0.001^*^
**Monthly income (CNY)**					
<999 (ref)					
1000-2999	−1.001	0.949	−1.055	(−2.862~0.860)	0.292
3000-5999	1.843	0.930	1.981	(0.018~3.668)	0.048^*^
≥6000	1.677	1.243	1.349	(−0.762~4.115)	0.178
**Family members with ADRD**					
No (ref)					
Yes	−2.681	1.328	−2.019	(−5.286~−0.076)	0.044^*^
**Knowledge training experience related to ADRD**					
No (ref)					
Yes	2.992	.859	3.484	(1.307~4.676)	0.001^*^

B: unstandardized coefficients, SE: Standard Errors, 95%CI: 95% Confidence Intervals, CNY: Chinese Yuan, ref: reference, ADRD: Alzheimer’s Disease and Related Dementias, ^*^: statistical difference.

## Discussion

In current research, the knowledge level of AD among residents in Jiulongpo District is relatively low. The scores obtained in this study are slightly inferior to those from a research project that surveyed representative cities and villages in the east, west, south, and north of China [13]. And also fall below the results of another study targeting the adult population in Zhuhai, China [19]. Notably, residents had particular difficulties recognizing AD symptoms and understanding caregiving needs, which highlights a critical gap in public awareness. This aligns with the results of surveys conducted by Sun et al. in Zhuhai, China [19], and Ma et al. in Jiaxing, China [20]. In summary, residents in Jiulongpo District have a relatively low level of understanding of AD, and there is significant room for improvement in each subdomain. Therefore, in the future, efforts should be intensified to promote AD knowledge and training programs in this region, with particular emphasis on the common symptoms of ADRD and the care of ADRD patients.

The survey found that the DAS scale scores of the interviewed residents were at a moderate level. Compared to the findings of Deng et al.‘s study [21], the residents participating in this study had a more positive attitude towards dementia and dementia patients. However, when compared to the results of Lu et al.’s study [13], Abdalrahim et al.’s research [22], and Aljezawi’s investigation [23], the residents’ perceptions and attitudes toward dementia patients appeared to be slightly more pessimistic. The low scores in the subdomain of “Positive social comfort” subdomian suggested that the surveyed residents may hold negative attitudes towards social interactions with dementia patients or feel anxious, uneasy, or lack confidence when facing them. Additionally, the low scores observed in the “Negative social comfort” subdomain could indicate that individuals experience more negative emotions or feelings of discomfort when interacting with dementia patients. Overall, the DAS scores of the surveyed residents were at a moderate level, leaving significant room for improvement. In the future, efforts can be made to cultivate a friendly attitude toward dementia by enhancing the public’s skills for positive interactions with dementia patients [24].

Interestingly, this study did not find significant differences in residents’ knowledge about Alzheimer’s Disease (AD) based on whether they had family members with AD or caregiving experience. Similarly, no significant differences were observed in their attitudes towards AD concerning whether they had caregiving experience. The findings of Dong et al. were consistent with this [25]. However, there is currently a lack of additional research to elucidate the reasons behind these observations. This study suggests that a possible reason is that although a few residents had family members with ADRD, they did not participate in their care, thus having little understanding of the disease. Those who had experience in caring for ADRD patients may not have received professional care training from specialized institutions or personnel and lacked social support [26]. As a result, they may have experienced significant psychological stress and emotional burdens during the caregiving process [26, 27], making it difficult for them to concentrate on learning and understanding ADRD-related knowledge. Furthermore, caregivers with heavy caregiving burdens may also experience associated stigma [28], which could lead to them not holding a more positive attitude towards ADRD patients. This survey recommends the following measures: through health education and training, family members should be equipped with basic knowledge about ADRD, including disease progression, treatment methods, and daily care skills [29]. Provide ADRD caregivers with knowledge and skill training related to ADRD care, while also offering psychological counseling to caregivers and providing them with more emotional support and social resources [30].

Our analysis also revealed that healthcare workers, urban residents, and individuals with prior dementia knowledge training exhibited better knowledge and more positive attitudes. Studies by scholars such as Zhao [31] and Wang [32] also showed that individuals with training in dementia-related knowledge had more knowledge about dementia and higher dementia attitude scores. Based on the evidence, educational and medical resources are relatively scarce in rural areas [33], while urban areas are richer in resources and have more diverse channels for accessing health information [34], making it easier for urban residents to access accurate information about ADRD and develop more favorable attitudes. Educational level is an important predictor of increased AD knowledge and favorable attitudes. Residents with higher education tend to exhibit better knowledge levels and more positive attitudes towards AD. Individuals who have received higher levels of education may develop a better understanding of complex health issues [19], which in turn helps to improve their awareness of AD and foster a more favorable attitude towards dementia [35–37]. We recommend that health education efforts should focus on rural areas, particularly on developing promotional materials tailored for residents with lower educational levels. These materials should primarily consist of easy-to-understand, engaging, and informative brochures and educational videos to improve the knowledge and attitudes of this population.

An unexpected finding was that residents with family members suffering from dementia displayed more negative attitudes. Direct caregiving or witnessing a family member with ADRD may impose psychological pressure or negative emotions on residents, leading them to hold a more negative attitude towards dementia. Therefore, for families with ADRD patients, psychological support and caregiving skills training should be provided to alleviate the caregiving burden and reduce social prejudices, helping them better cope with this challenge. This not only contributes to improving the mental health of family members and caregivers but also enhances the quality of care and life for ADRD patients, achieving a win-win outcome.

This study also has several limitations that need to be considered. Firstly, it employed a cross-sectional survey design, which means it cannot determine causality but only establish associations between variables at a specific point in time. Secondly, the questionnaire design did not control for all potential confounding factors that could influence the results, such as smoking, alcohol consumption, and lifestyle. Thirdly, the survey results were based on subjective responses from participants, which may have been influenced by social desirability, leading to more positive answers and potential information bias. These limitations need to be improved in future research.

## Conclusion

Adult residents in Jiulongpo District, Chongqing, have limited understanding of AD, particularly in terms of its symptoms and care, which may hinder early detection and management control of dementia. Additionally, their scores on the DAS are at a moderate level, indicating significant room for improvement. We recommend incorporating dementia-related knowledge and fostering a friendly attitude towards it into the education system. Furthermore, efforts should be intensified to conduct public health education on the prevention, early identification, and care of ADRD, cultivating a friendly attitude towards dementia patients, and enhancing support for caregivers. These measures will not only improve the public’s ability to identify dementia early but also reduce social prejudices and promote social harmony. Such initiatives are of great significance in addressing the increasingly severe public health challenge posed by dementia.

## Supporting information

S1 Dataset Data on Cognition and Attitudes towards Alzheimer’s Disease among Adult Residents in Jiulongpo District, Chongqing City.
(XLSX)

## Abbreviations

AD: Alzheimer’s disease;
ADKS: Alzheimer’s disease knowledge scale
DAS: Dementia attitudes scale
ADRD: Alzheimer’s disease and related dementias
WHO: World health organization
M: Mean
SD: Standard deviation
B: Unstandardized coefficients
SE: Standard errors
95%CI: 95% Confidence intervals.
